# T- and B-Cell-Mediated Protection Induced by Novel, Live Attenuated Pertussis Vaccine in Mice. Cross Protection against Parapertussis

**DOI:** 10.1371/journal.pone.0010178

**Published:** 2010-04-15

**Authors:** Pascal Feunou Feunou, Julie Bertout, Camille Locht

**Affiliations:** 1 Inserm U1019, Lille, France; 2 Institut Pasteur de Lille, Lille, France; 3 CNRS UMR8204, Lille, France; 4 Univ Lille Nord de France, Lille, France; 5 IFR142, Molecular and Cellular Medicine, Lille, France; Institut Pasteur, France

## Abstract

**Background:**

Despite the extensive use of efficacious vaccines, pertussis still ranks among the major causes of childhood mortality worldwide. Two types of pertussis vaccines are currently available, whole-cell, and the more recent acellular vaccines. Because of reduced reactogenicity and comparable efficacy acellular vaccines progressively replace whole-cell vaccines. However, both types require repeated administrations for optimal efficacy. We have recently developed a live attenuated vaccine candidate, named BPZE1, able to protect infant mice after a single nasal administration.

**Methodology/Principal Findings:**

We determined the protective mechanism of BPZE1-mediated immunity by using passive transfer of T cells and antibodies from BPZE1-immunized mice to SCID mice. Clearance of *Bordetella pertussis* from the lungs was mediated by both BPZE1-induced antibodies and CD4^+^, but not by CD8^+^ T cells. The protective CD4^+^ T cells comprised IFN-γ-producing and IL-17-producing subsets, indicating that BPZE1 induces both Th1 and Th17 CD4^+^ T cells. In addition, and in contrast to acellular pertussis vaccines, BPZE1 also cross-protected against *Bordetella parapertussis* infection, but in this case only the transfer of CD4^+^ T cells conferred protection. Serum from BPZE1-immunized mice was not able to kill *B. parapertussis* and did not protect SCID mice against *B. parapertussis* infection.

**Conclusions/Significance:**

The novel live attenuated pertussis vaccine BPZE1 protects in a pre-clinical mouse model against *B. pertussis* challenge by both BPZE1-induced antibodies and CD4^+^ T cell responses. It also protects against *B. parapertussis* infection. However, in this case protection is only T cell mediated.

## Introduction

*Bordetella pertussis* is the main etiological agent of whooping cough or pertussis, an acute respiratory disease with increasing prevalence and incidence, particularly in neonates [1], [2]. Despite the extensive use of efficacious vaccines, *B. pertussis* still represents a major global public health problem and one of the top 10 causes of childhood mortality [3]–[5]. Two types of pertussis vaccines are currently available [6]. The first generation vaccines consist of killed whole *B. pertussis* cells (wcPV) and have shown up to over 90% protective efficacy [7]. However, these vaccines have been associated with local and systemic side-effects, including local swelling, high fever and, in rare cases, encephalopathy and even death. These drawbacks have led to the development of new-generation, acellular vaccines (aPV). Initially developed and used in Japan, they consist of highly purified protective antigens [8]–[10]. Although the aPV have been shown to be much less reactogenic than the wcPV, three vaccine injections are still needed for optimal protection, and the protective efficacy of the best aPV has consistently been lower than that of the best wcPV [6], [11]. Furthermore, the production of aPV is much more expensive than that of wcPV, making them less affordable for developing countries. None of the currently available vaccines targets mucosal immunity, although *B. pertussis* is a mucosal pathogen, and the infection strictly confined to the upper respiratory tract. Mucosal immunity could therefore conceivably contribute to protection [12].

In addition to *B. pertussis*, *Bordetella parapertussis*, a closely related species sharing many of the *B. pertussis* virulence factors, can also cause a whooping cough-like disease. Both pathogens can be found in the same infected host at the same time [13], [14]. Reported frequencies of whooping cough caused by *B. parapertussis* range from 2 to 36% [15], [16]. However, *B. parapertussis* infections are probably underestimated, most likely because the disease is milder than that caused by *B. pertussis* [17], [18]. Cross-protection conferred by existing *B. pertussis* vaccines, especially aPV, against *B. parapertussis* infection is very poor [19]. It has recently been shown that one of the reasons for this poor cross-protection is the presence of the O antigen on the surface of *B. parapertussis*, which allows this organism to escape anti-*B. pertussis* antibody-mediated immunity [20].

We have recently constructed BPZE1, a live attenuated *B. pertussis* vaccine strain, resulting from the genetic inactivation or removal of three major virulence factors, tracheal cytotoxin, pertussis toxin (PTX) and dermonecrotic toxin, as described in detail in [21]. Athough BPZE1 does not produce tracheal cytotoxin and dermonecrotic toxin, it still produces immunogenic PTX, albeit in a genetically detoxified form. This vaccine strain is highly protective against *B. pertussis* challenge in mouse models and showed genetic stability during *in vivo* or *in vitro* passages [22]. Interestingly, BPZE1 also conferred significant cross-protection against *B. parapertussis* infection [21]. In this study, we investigated the mechanisms underlying the protective immunity induced by BPZE1 against *B. pertussis* and *B. parapertussis* by using adoptive transfer to severe combined immunodeficiency (SCID) mice.

## Results

### *B. pertussis* causes persistent infection in SCID mice

In immuno-competent mice, infection with 10^5^ to 10^6^ virulent *B. pertussis* results in a typical increase of the bacterial burden by a factor of 10 during the first 7 days, followed by a general decline with a total clearance of the bacteria at day 30 after infection [21], [23]. Extra-pulmonary disseminated *B. pertussis* infection is rarely seen, both in mice [24] and in humans [25]. This colonisation profile shows control of the bacteria resulting probably from a combination of both innate and adaptive immune responses [23].

**Figure 1:**
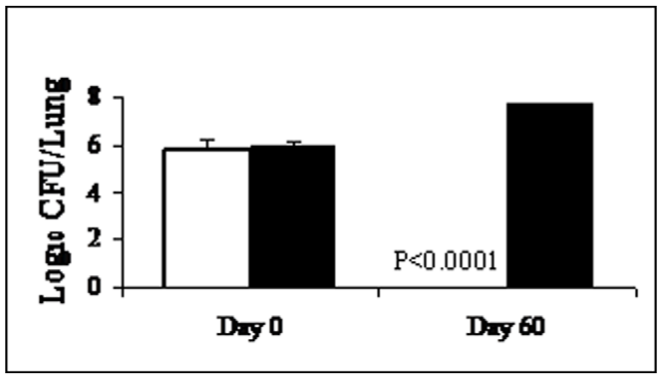
*B. pertussis* causes persistent respiratory infection in SCID mice. Two groups (BALB/c, white columns; and SCID, black columns) of 5 mice were infected i.n. with ∼1×10^6^ *B. pertussis* BPSM and sacrificed 3 h (Day 0) or eight weeks later (Day 60) for CFU counts in the lungs. The results are expressed as means of log CFU values per lung (± standard error) and are representative of three independent experiments.

Immuno-compromised mice, deficient in B and/or T cell responses fail to clear *B. pertussis* infection [26]–[29] and may therefore constitute good in-vivo models to address the effector mechanisms of adaptive immunity to *B. pertussis* infection. In order to investigate the role of the BPZE1-induced adaptive immune responses in the clearance of *B. pertussis*, we therefore used SCID mice defective in T, B and natural killer (NK) cell populations. First, the mice were intranasally (i.n.) infected with *B. pertussis* BPSM [30], and the numbers of viable bacteria recovered from the lungs were determined at different time points. As shown in Fig. 1, three hours (Day 0) and eight weeks (Day 60) after infection, *B. pertussis* BPSM organisms were present in the lungs of the SCID mice. Whereas all the bacteria were essentially cleared in the immuno-competent mice 8 weeks after challenge, the SCID mice contained roughly 10 fold more bacteria at 8 weeks after challenge compared to day 0, similar to what is usually seen in immuno-competent mice at day 7 post-infection [21], [22]. These results are consistent with the fact that adaptive immunity is required for the clearance of *B. pertussis* from the mouse respiratory tract [23], and allow us to use SCID mice as a suitable model to identify the effector mechanisms of adaptive immunity induced by BPZE1 against *B. pertussis*.

### Adoptive transfer of spleen cells or serum from BPZE1-vaccinated mice protects SCID mice against *B. pertussis*

**Figure 2:**
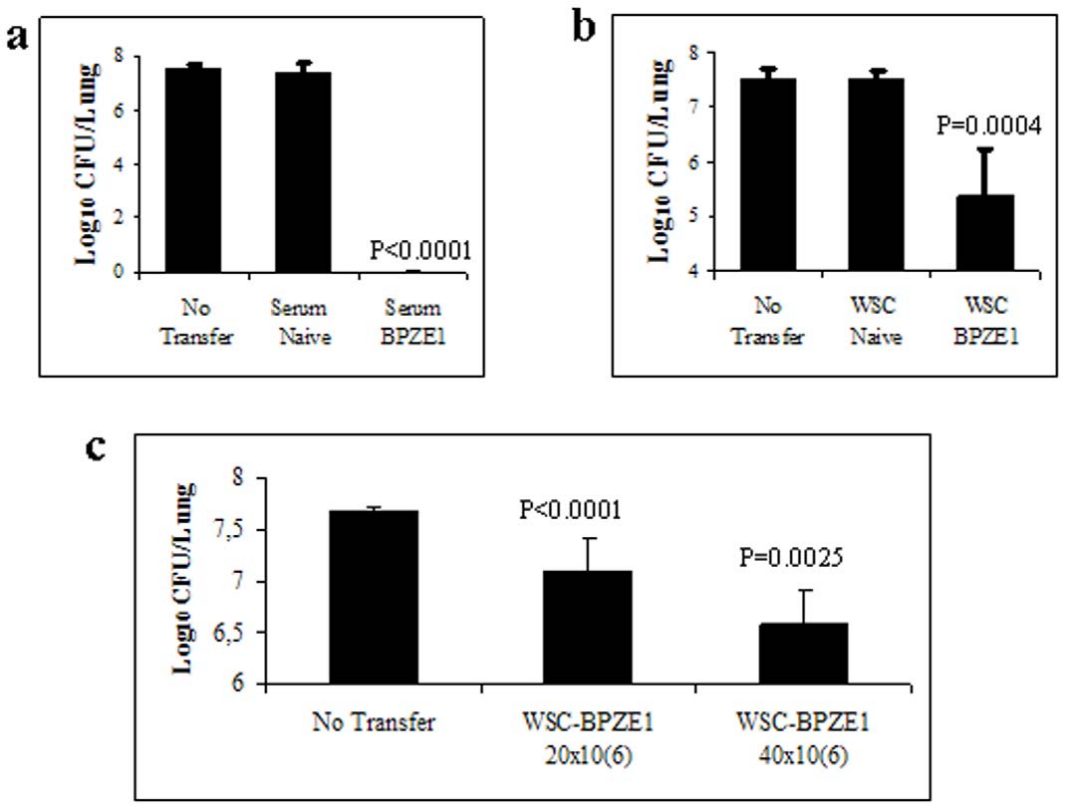
Transfer of protection by spleen cells and serum from BPZE1-immunized mice. 100 µl of serum (**a**) or 50×10^6^ whole spleen cells (WSC) (**b**) or indicated amounts of WSC (**c**) from non-immunized (Naïve) or BPZE1-immunized (BPZE1) BALB/c mice were transferred intraperitoneally to SCID mice 24 h before they were intranasally infected with virulent *B. pertussis* BPSM (1×10^6^ CFU). Non-transferred SCID mice (No transfer) served as controls. The mice were sacrificed 7 days after challenge, and CFUs in the lungs were counted. The results are expressed as means of log CFU values per lung from 5 mice per group (± standard error) and are representative of three independent experiments.

SCID mice were thus used to study the contribution of T cells and antibodies in the protection conferred by BPZE1 against *B. pertussis* using transfer experiments. Either serum (100 µl) or whole spleen cells (50×10^6^) from non-immunized or mice immunized with BPZE1 eight weeks previously were transferred to groups of five female SCID mice. Prior to transfer we verified that the BPZE1-vaccinated mice had produced *B. pertussis*-specific T and B cells (data not shown). This was done by using *B. pertussis* antigen-specific T cell proliferation assays and by measuring anti-*B. pertussis* antibodies in the serum, as described [21]. 24 hours after transfer, the SCID mice were i.n. infected with 10^6^ *B. pertussis* BPSM and sacrificed 7 days later to determine the numbers of colony-forming units (CFU) in the lungs. As shown in Fig. 2a, transfer of 100 µl serum from BPZE1-immunized BALB/c mice conferred strong protection, as no bacteria were detected in the lungs within 7 days after challenge. No protection was achieved with serum transfer from non-immunized mice, as at day 7 post infection, the bacterial burden (∼10^7^) was similar to that observed in the infected control mice. In addition to the serum, the transfer of spleen cells from BPZE1-immunized mice also conferred a significant level of protection to SCID mice against infection with *B. pertussis*, as evidenced by a reduction by approximately 2 logs in CFU compared to non-transferred mice or to SCID mice that had received spleen cells from non-vaccinated donors (Fig. 2b). In a parallel experiment, the levels of protection increased with increasing amounts of T cells transferred (Fig. 2c). These data thus demonstrate that both B and T cells are involved in BPZE1-mediated protection.

### Role of CD4^+^ and CD8^+^ T cells in BPZE1-mediated immunity

**Figure 3:**
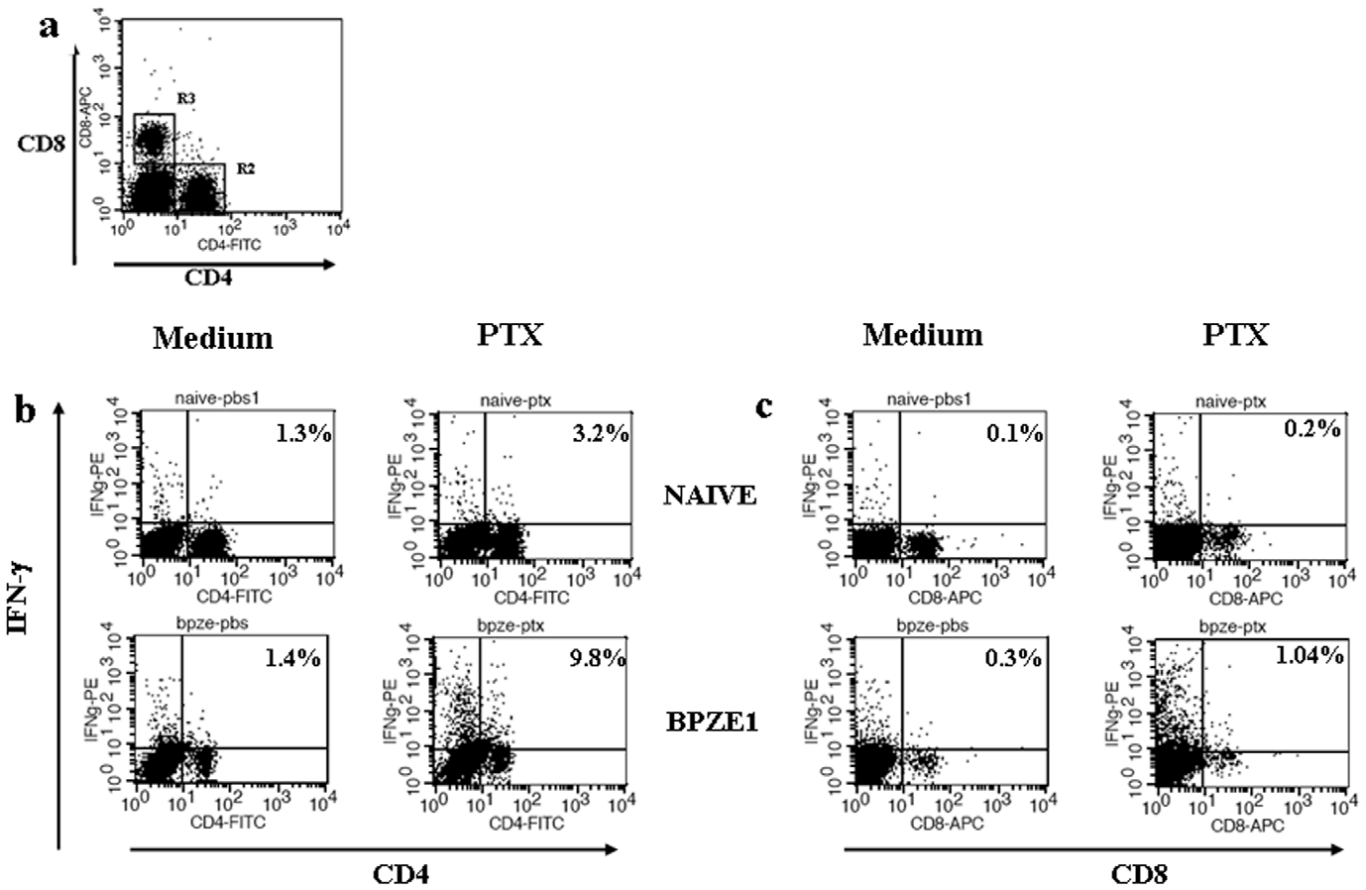
CD4^+^ T phenotype of IFN-γ-producing T cells following BPZE1 administration. BALB/c mice were left untreated (Naïve) or immunized intranasally with 10^6^ CFU of *B. pertussis* BPZE1 (BPZE1). Eight weeks later, the mice were sacrificed and spleens of three individual mice per group were pooled and homogenized to obtain single-cell suspensions. 2.5×10^6^ cells were cultured in triplicate overnight in the presence of 10 µg/ml PTX (PTX) or left unstimulated (Medium). Spleen-derived T cells were then labelled for surface expression of CD4 and CD8 (**a**), and intracellular IFN-γ expression by CD4^+^ (**b**) and CD8^+^ (**c**) T cells was assessed by flow cytometry. The results shown are representative of three independent experiments.

We have previously shown that BPZE1 vaccination induces both *B. pertussis*-specific antibodies and IFN-γ, whereas it induces only low levels of Th2 cytokines [21]. Several studies have pointed to an important role of IFN-γ in the control against *B. pertussis* infection [24], [27], [29]. Since IFN-γ can be produced by different cell types, we wanted to know whether either the CD4^+^ or the CD8^+^ T cells are responsible for *B. pertussis*-specific IFN-γ production upon vaccination with BPZE1. The splenocytes of non-immunized and of BPZE1-immunized mice were isolated 8 weeks after vaccination and stimulated *in vitro* with PTX (10 µg/ml) or filamentous hemagglutinin (FHA, 5 µg/ml) for 24 hours. Their surface expression of CD4 and CD8 (Fig. 3a), as well as their intracellular IFN-γ production were then analyzed by flow cytometry. As shown in Fig. 3b, most of the PTX-specific IFN-γ was produced by the CD4^+^ or the CD4^−^/CD8^−^ T cells from BPZE1-immunized mice, whereas the CD8^+^ T cells did not contribute to the PTX-specific IFN-γ response at a significant level (Fig. 3c). In fact, most of the IFN-γ was produced by the CD4^−^/CD8^−^ T cells, most likely NK or NKT cells. As expected, neither CD4^+^ nor CD8^+^, nor CD4^−^/CD8^−^ T cells from non-vaccinated mice produced PTX-specific IFN-γ. Similar results were obtained when FHA was used (data not shown).

**Figure 4:**
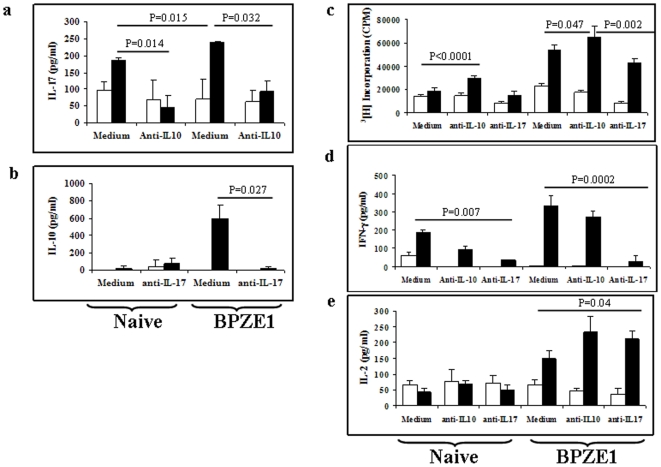
Cytokine production profile of splenocytes from BPZE1-immunized mice. BALB/c mice were left untreated (Naïve) or immunized intranasally with 10^6^ CFU of *B. pertussis* BPZE1 (BPZE1). Eight weeks later, the mice were sacrificed, and spleen cells were stimulated in triplicate in the presence (black bars) or absence (white bars) of 10 µg/ml FHA, with or without (Medium) 10 µg/ml anti-IL-10 or anti-IL-17 antibodies. After 60 h of culture, the secretion of IL-17 (**a**), IL-10 (**b**), IFN-γ (**d**) and IL-2 (**e**) in the culture supernatants was determined by sandwich ELISA, and the proliferation was assessed by [^3^H] thymidine incorporation (**c**). The results are the expressed as mean values (± standard error) for triplicate cultures from four mice per group and are representative of three experiments.

More recently, IL-17-producing Th17 cells have been proposed to participate in protective cellular immunity against *B. pertussis* [31], and PTX promotes the generation of IL-17- producing CD4^+^ T cells [32]. On the other hand, FHA, present on the surface of BPZE1 [21], has been shown to induce the production of IL-10 [33], a cytokine produced by type 1 regulatory T (Treg) cells and serving to evade the protective Th1 responses. Since it was not known whether administration of BPZE1 induces IL-17 or IL-10 *in vivo*, we stimulated spleen cells from BPZE1-vaccinated mice and from naïve control mice with FHA and measured the production of these two cytokines in the culture supernatants. High amounts of both IL-10 and IL-17 were produced upon FHA stimulation by spleen cells from BPZE1-vaccinated and naïve mice. The addition anti-IL-10 antibodies significantly decreased IL-17 secretion, suggesting a positive contribution of IL-10 on IL-17 production (Fig. 4a). In contrast to IL-17, the FHA-induced IL-10 production was much higher in the spleen cells from BPZE1-immunized mice than from the control animals. Blocking IL-17 by using anti-IL-17 antibodies completely abrogated IL-10 secretion (Fig. 4b). These data show that IL-10 is an essential factor for IL-17 production and reciprocally, and suggest a collateral development pathway between IL-17-producing Th17 cells and IL-10-producing Treg cells.

We next examined *in vitro* the effects of blocking anti-IL-10 and anti-IL-17 on the proliferation and other cytokines produced by FHA-stimulated spleen cells. Incubation of whole spleen cells from BPZE1-immunized mice with FHA induced stronger proliferation and higher IFN-γ and IL-2 amounts than those from naive littermates (Fig. 4c–e). The addition of blocking anti-IL-10 antibodies to the culture slightly increased proliferation, had no effect on the IFN-γ production, but increased IL-2 production in the BPZE1-immunized group (Fig. 4c–e). When anti-IL-17 antibodies were added to the culture, proliferation was slightly inhibited (Fig. 4c), whereas the IFN-γ production strongly inhibited (Fig. 4d), and the IL-2 production was augmented (Fig. 4e). Thus, proliferation appeared to be inversely regulated by IL-10 and IL-17.

**Figure 5:**
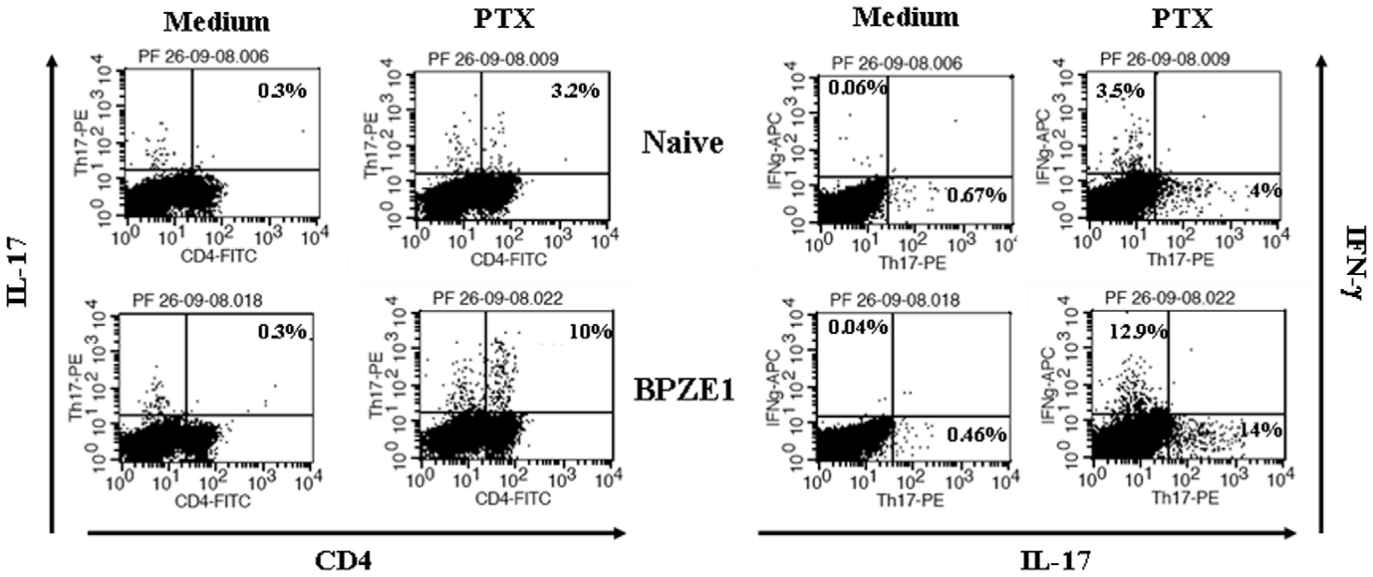
IFN-γ and IL-17 are produced by distinct *B. pertussis*-specific CD4^+^ T cell subsets from BPZE1-immunized mice. BALB/c mice were left untreated (Naïve) or immunized intranasally with 10^6^ CFU of *B. pertussis* BPZE1 (BPZE1). Eight weeks later, the mice were sacrificed, and spleens of three individual mice per group were pooled and homogenized to obtain single-cell suspensions. 2.5×10^6^ cells were cultured in triplicate overnight in the presence (PTX) or absence (Medium) of 10 µg/ml PTX. Spleen-derived T cells were then labelled for surface expression of CD4, and intracellular IFN-γ and IL-17 expression was assessed by flow cytometry. The results are representative of three independent experiments.

**Figure 6:**
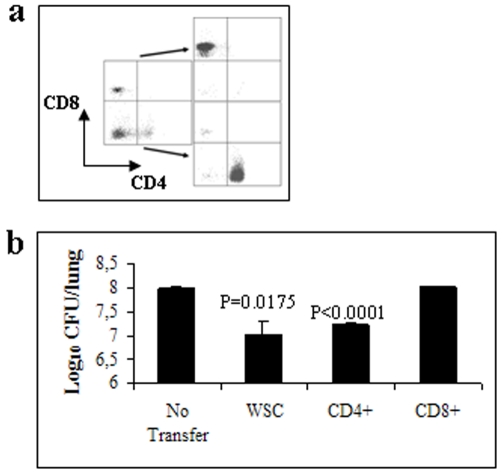
Protection by purified CD4^+^ T cells from BPZE1-immunized mice. Purified CD4^+^ or CD8^+^ T cells from BPZE1-immunized BALB/c mice (**a**) were transferred intraperitoneally to naïve SCID mice 24 h before they were intranasally infected with virulent *B. pertussis* BPSM. The mice were sacrificed 8 days later, and CFUs present in the lungs were counted (**b**). The results are expressed as means of log CFU values per lung from 5 mice per group (± standard error) and are representative of two independent experiments.

Since BPZE1 administration resulted in the production of both Th1 and Th17 CD4^+^ T cell-mediated *B. pertussis*-specific IFN-γ and IL-17, respectively, we investigated whether it is the BPZE1-induced CD4^+^ T cell population that confers protection in the SCID mouse model. As shown in Fig. 5 (left panel), BPZE1 immunization induced PTX-specific CD4^+^ IL-17-producing T cells. Importantly, the IL-17-producing T cells were different from the IFN-γ-producing cells (Fig. 5, right panel), and we did not observe IFN-γ-, IL-17- double positive CD4^+^ T cells. CD4^+^ and CD8^+^ T cells were thus purified from BPZE1-immunized mice (Fig. 6a) using a cell sorter separation technology, as described in Materials and Methods, and then transferred to SCID mice 24 h prior to *B. pertussis* BPSM challenge. As illustrated in Fig. 6b, 7.5×10^5^ purified CD4^+^ T cells from BPZE1-immunized mice transferred to SCID mice provided significant protection against *B. pertussis* challenge, whereas the transfer of the same amount of CD8^+^ T cells did not. Thus, both the *in vitro* and the *in vivo* results indicate that within the T cell compartment, the CD4^+^ T cells play a major role in the BPZE1-induced protective immunity, most likely through their ability to secrete antigen specific IFN-γ and IL-17.

### BPZE1-induced cellular but not humoral immunity cross-protects against *B. parapertussis*

**Figure 7:**
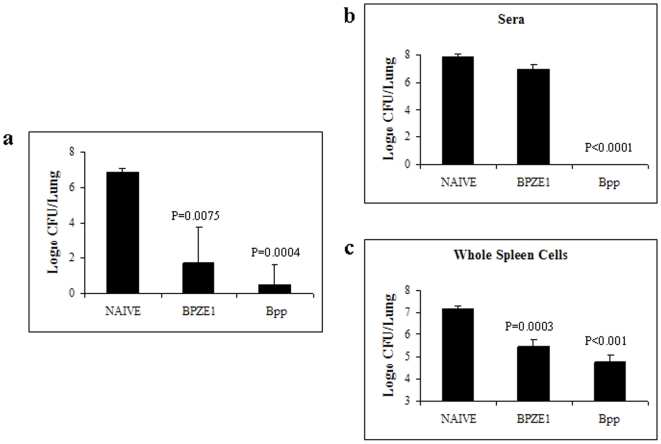
BPZE1-induced immunity cross-protects against *Bordetella parapertussis*. BALB/c mice were intranasally infected with 1×10^6^ CFU of *B. pertussis* BPZE1 (BPZE1) or *B. parapertussis* (Bpp) or left untreated (Naïve). The mice were then challenged 8 weeks later with 1×10^6^ CFU *B. parapertussis* and sacrificed 7 days after challenge for counting of CFUs in the lungs (**a**). 100 µl of sera (**b**) or 50×10^6^ whole spleen cells (**c**) from BPZE1-immunized, *B. parapertussis*-infected or naïve mice were injected intraperitoneally into naive SCID mice. 24 h later, the mice were intranasally infected with *B. parapertussis* and sacrificed seven days later for CFU counts in the lungs. The results are expressed as means of log CFU values per lung from 5 mice per group (± standard error) and are representative of three independent experiments.

Although wcPV provides some level of protection against *B. parapertussis* infections in mouse models, aPV does not, but instead enhances colonisation by *B. parapertussis* [19]. In contrast, we have previously shown that administration of BPZE1 protects mice against *B. parapertussis* [21]. To identify the BPZE1-induced immune effector functions that provide cross-protection against *B. parapertussis*, we transferred antisera or spleen cells from BPZE1-immunized mice to SCID mice prior to *B. parapertussis* challenge. First, we confirmed that BPZE1 provides cross-protection against *B. parapertussis*. As shown in Fig. 7a, BPZE1-immunized mice or animals infected with *B. parapertussis* 8 weeks prior to re-infection showed substantially reduced CFU counts in the lungs, in comparison to non-immunized but challenged control mice. Groups of five SCID mice were then injected with either 100 µl of serum or 50×10^6^ whole spleen cells from non-immunized, BPZE1-immunized or *B. parapertussis*-infected BALB/c mice 8 weeks previously. One day later the SCID mice were challenged with *B. parapertussis* and sacrificed 7 days post-challenge to determine bacterial counts in the lungs. The transfer of serum from non-immunized or BPZE1-immunized BALB/c mice had no significant effect on colonization by *B. parapertussis* during the first 7 days post-challenge (Fig. 7b), whereas, transferring *B. parapertussis* immune serum to recipient SCID mice resulted in total clearance of the bacteria from the lungs at day 7. Unlike the serum, transfer of spleen cells from BPZE1-immunized mice resulted in significant protection of the SCID mice against *B. parapertussis* infection, although slightly lower than that obtained by the transfer of spleen cells from *B. parapertussis*-infected mice (Fig. 7c). BPZE1-induced cross-protection against *B. parapertussis* is thus essentially T-cell mediated, while immunity induced by *B. parapertussis* infection elicits both protective B and T cells responses.

### Serum from BPZE1-vaccinated mice is able to kill *B. pertussis* but not *B. parapertussis*

**Figure 8:**
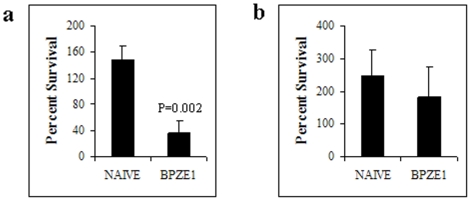
Survival of *B. pertussis* and *B. parapertussis* in the presence of serum from non-immune or BPZE1-immunized mice. 300 CFU of *B. pertussis* (**a**) and *B. parapertussis* (**b**) were incubated at 37°C for 1 h in the presence of 100 µl of 80% serum from unimmunized (Naïve) or BPZE1-immunized (BPZE1) mice and then plated onto Bordet Gengou blood agar plates for CFU counting. The results are presented as percent survival relative to that of a PBS control (in the absence of serum) (± standard error) for 3 mice per group, evaluated on 3 different plates per mouse, and are representative of three independent experiments.

The failure of serum from BPZE1-immunized mice to protect SCID mice against *B. parapertussis* infection suggests that it is unable to kill *B. parapertussis* bacteria. We thus carried out a serum killing assay to compare survival of *B. parapertussis* with that of *B. pertussis* after incubation with serum from naïve or BPZE1-immunized mice. 300 CFU of *B. pertussis* or *B. parapertussis* were incubated with serum from naïve or BPZE1-immunized mice for 1 h at 37°C. The bacteria were then plated on BG-blood agar for CFU counting. Significant *B. pertussis* killing was observed by serum from BPZE1-immunized mice compared to serum from non-immunized mice (Fig. 8a), whereas the BPZE1 serum did not significantly reduce the amounts of *B. parapertussis* bacteria compared to serum from non-immunized mice (Fig. 8b), indicating that the serum from BPZE1-immunized mice has no antimicrobial activity against *B. parapertussis* although it is active against *B. pertussis*.

## Discussion

In this study, we used SCID mice, deficient in B, T and NK T cells, to examine the mechanisms of protective immunity against *B. pertussis* and *B. parapertussis* i.n. infections induced by the recently developed live attenuated vaccine strain BPZE1. Although the mouse model has its obvious limitations, it is the most widely used animal model to study pertussis vaccines, and there has been a good correspondence between vaccines that protect children against pertussis and those that protect mice against i.n. infection with *B. pertussis* [34]. We found that, unlike immuno-competent mice, SCID mice are unable to clear *B. pertussis* infection, thus qualifying them as a suitable animal model to study the contribution of the different effectors molecules and cells in the protective adaptive immunity against *Bordetella*. Previous studies using gene knock-out mice with deficiencies for specific immune functions, including Th1-type cytokines and immunoglobulins, have already shown the importance of both B cells and CD4^+^ T cells in immunity against *B. pertussis* [23], [26]–[29], [34]. As a complement to these studies, transfer experiments to immuno-deficient mice, such as SCID mice, enable us to identify molecules and cell types that exert direct effectors functions against the invading pathogen. We therefore used that technology to identify the effectors molecules or cells involved in BPZE1-induced protection. In this model, both serum-free T cells and anti-BPZE1 antisera were able to provide significant protection against *B. pertussis* infection, indicating that both antibodies and T cells contribute to BPZE1-induced protection and that either one can confer protection. The mechanisms of immunity induced by BPZE1 are thus comparable to that induced by infection with virulent *B. pertussis*.

Although we have so far tested only one *B. pertussis* challenge strain and other, in particular recent, circulating clinical isolates need to be tested in future studies, it is likely that BPZE1 protects against a variety of *B. pertussis* strains, since nasal immunization with BPZE1 even protects against *B. parapertussis* infection [21]. The ability of pertussis vaccines to protect against *B. parapertussis* has remained controversial. Several epidemiological and animal vaccine studies have shown poor cross-protection of *B. pertussis* vaccines against *B. parapertussis*, especially when aPV are used [15], [19]. However, infection with *B. pertussis* has been reported to protect mice against *B. parapertussis* infection, and vice versa [35], [36]. Since nasal vaccination with BPZE1 mimics much closer *B. pertussis* infection than systemic immunization with aPV, the cross-protective results observed upon BPZE1 administration are consistent with the previously observed reciprocal immunity induced by infection [36].

Interestingly, only the transfer of T cells from BPZE1-immunized mice to SCID mice provided protection against *B. parapertussis* infection, indicating that the BPZE1-induced cross-protection is cell-mediated. However, this does not rule out that other effector mechanisms may also contribute to protection against *B. parapertussis*. In contrast, BPZE1-induced antibodies did not protect against *B. parapertussis* infection. This was confirmed by the fact that BPZE1-induced antibodies were not able to kill *B. parapertussis*, whereas they were efficient in killing *B. pertussis*. These findings are consistent with recent reports showing that, although anti-*B. pertussis* antibodies cross-react with *B. parapertussis* antigens, *B. parapertussis* displays an O antigen that is not produced by *B. pertussis*, and that inhibits binding of the antibodies to the bacterial cell surface [20] and protects the bacteria from complement-mediated killing by inhibiting complement C3 deposition on the bacterial surface [37]. The presence of O antigen on the surface of *B. parapertussis* may thus explain why BPZE1-induced cross-protection is solely T-cell mediated. Unlike protection resulting from infection [38], aPV-induced protection against pertussis is essentially antibody mediated [34]. It is therefore not surprising that vaccination with aPV offers little protection against *B. parapertussis*.

Considering the importance of T cell-mediated immunity in the protection against *B. pertussis* and *B. parapertussis*, we analyzed the BPZE1-induced T cell responses in more detail. Previous studies had already shown that vaccination with BPZE1 induces a strong Th1 response in mice, as evidenced by high levels of antigen-specific IFN-γ and low IL-5 responses [21]. BPZE1-induced antigen-specific IFN-γ was confirmed here, and we further show that BPZE1 also induces antigen-specific IL-17. This cytokine has previously been shown to be associated with protection against *B. pertussis* via activation of *B. pertussis* killing by macrophages, and to act in concert with IFN-γ although probably through distinct mechanisms [32]. Both IFN-γ and IL-17 were produced by *B. pertussis* antigen-specific CD4^+^ T cells in BPZE1-immunized mice, although, and as expected, by two different CD4^+^ T cell sets, indicating that in addition to antibodies, BPZE1 vaccination also induces Th1 and Th17 cells. We did not find CD4^+^ IL17^+^ IFNγ^+^ T-cell in our model. Lack of double positive has also been reported in other studies on mice [39], [40] and humans [41], [42], although human CD4^+^ T cells have been shown to be able to secrete both IL-17 and IFN-γ under some circumstances [43], [44]. We also detected significant amounts of IL-17 and IFN-γ secretion by FHA stimulated-spleen cells from naïve mice, although the cytokine amounts were higher in the BPZE1-vaccinated mice than in the naïve animals. It has been shown by Higgins et al. [31] that FHA-stimulated spleen cells from mice vaccinated with pertussis vaccines produce IL-17 and IFN-γ, while naïve mice do not. These discrepancies may be due to the differences in the amounts of FHA used to stimulated the spleen cells (10 µg/ml used here, compared to 2 µg/ml used by [31]). Transfer of purified CD4^+^ T cells comprising both the Th1 and the Th17 subsets, but not of CD8^+^ T cells protected the SCID mice against *B. pertussis* infection.

In addition to Th1-type cytokines and IL-17, BPZE1 vaccination also induced IL-10, an anti-inflammatory and regulatory cytokine, previously reported to down-modulate Th-1 type responses and to help *B. pertussis* to evade protective immunity [33]. We found that neutralizing IL-10 by blocking antibodies indeed increased T cell proliferation in naïve and vaccinated mice, and IL-2 secretion in vaccinated mice upon stimulation with FHA *in vitro*, but inhibited IL-17 secretion in both groups. These observations are in disagreement with reports on human T cells, which have demonstrated that neutralization of IL-10 or TGF-β abrogated IL-17 production, while it increased IFN-γ production by PBMC from Hepatitis C virus-infected patients [44]. However, although the mechanism of this IL-10-dependent induction of IL-17 remains to be investigated, our observation is consistent with previous report [42] and with the results of a very recent study indicating that enzymatically inactive PTX induces IL-10 production by human monocyte-derived dendritic cells, which in turn enhances IL-17 production [45]. However, it has been proposed that the IL-10 effect on IL-17 production is indirect via its ability to fine tune regulatory cytokine expression. In addition to PTX, the *B. pertussis* lipopolysaccharide may also contribute to the induction of IL-10 and subsequently IL-17 [46]. The major triggering molecule may perhaps be identified through the use of defined *B. pertussis* mutants lacking the genes that code for PTX. The fact that neutralization of IL-10 had a much stronger effect on the reduction of IL-17 production than on the IFN-γ secretion supports the notion that both cytokines are produced by distinct T cell subtypes during *B. pertussis* infection [47].

Conversely, neutralizing IL-17 with blocking antibodies reduced IL-10 secretion in vaccinated mice and IFN-γ production both in naïve and in vaccinated mice, but increased IL-2 secretion in vaccinated mice. These findings suggest that IL-17 is an important cytokine not only for the induction of IFN-γ-producing Th1 cells, but also for IL-10 secretion after BPZE1 administration. The fact that blocking IL-17 increased IL-2 secretion, while decreasing IL-10 secretion, leads us hypothesize that IL-17 blocking did not act directly on IL-2 secretion, but perhaps indirectly via the inhibition of IL-10 secretion, known to be an inhibitor of IL-2 production. However we cannot exclude a direct blocking IL-17 effect on the increased IL-2 secretion. In naïve mice, neutralizing IL-10 or IL-10 and IL-17 did not affect IFN-γ and IL-2 secretion, respectively. Thus, it appears that even in naïve mice, IL-10 and IL-17 differentially modulated cytokine secretion.

Collectively, the data presented here show that a single nasal administration of the live attenuated *B. pertussis* BPZE1 vaccine strain is able to induce the broad spectrum of protective effector mechanisms associated with control of infection by human adapted *B. pertussis* species. Both B and CD4^+^ T cells contribute into BPZE1-induced protection against *B. pertussis*, while cross-protection against *B. parapertussis* in essentially T cells mediated. The BPZE1-induced T cells comprise the IFN-γ-producing Th1-type and Th17 cells, both likely to participate in the cell-mediated protection. The involvement of Th17 in the protective immunity against pertussis has only been very recently recognized, but may be particularly relevant in the light of the growing evidence that these cells play an important role in mucosal immunity, especially towards respiratory pathogens [48]. Since *B. pertussis* is a strictly respiratory pathogen, there is little doubt that future studies will clarify the role of Th17 cells in the protective mechanisms at the mucosal site that constitutes the port of entry of this important pathogen. In addition, the role of the *B. pertussis*-specific IFN-γ producing CD4^−^/CD8^−^ cells from BPZE1-immunized mice warrants further investigation. These cells are most likely NK or NKT cells. It has previously been demonstrated that *B. pertussis* infection induces high frequencies of IFN-γ-secreting NK cells and that these cells play an important role in protection, especially against disseminated *B. pertussis* infection [27]. In future studies we will thus address whether the BPZE1-induced IFN-γ-producing CD4^−^/CD8^−^ cells are NK cells that also may participate in protection against *B. pertussis* and *B. parapertussis* infection.

## Materials and Methods

### Ethics statement

All animal experiments were performed following the guidelines of the Institut Pasteur de Lille animal study board, which conforms to the Amsterdam Protocol on animal protection and welfare, and Directive 86/609/EEC on the Protection of Animals Used for Experimental and Other Scientific Purposes, updated in the Council of Europe's Appendix A (http://conventions.coe.int/Treaty/EN/Treaties/PDF/123-Arev.pdf). The animal work also complied with the French law (n° 87-848 dated 19-10-1987) and the European Communities Amendment of Cruelty to Animals Act 1976. All manipulations involving animals were carried out by qualified personnel. The animal house was placed under the direct control of the Institut Pasteur de Lille director who is the “designated responsible person” under French law. The study has been approved by Ethical Committee for experiments on animals of the region Nord-Pas-de-Calais (approval number AF 03/2009).

### *Bordetella* strains and growth conditions

The *B. pertussis* strains used in this study were streptomycin-resistant BPSM [30] and BPZE1 [21], both derived from *B. pertussis* Tohama I. The *B. parapertussis* strain was a streptomycin-derivative of strain 12822, kindly provided by N. Guiso, Institut Pasteur de Paris. All *Bordetella* strains were grown on Bordet-Gengou (BG) agar (Difco Laboratories, Detroit, Michigan, United States) supplemented with 1% glycerol, 20% defibrinated sheep blood, and 100 µg/ml streptomycin at 37°C. After growth, the bacteria were harvested by scraping the plates and resuspended in phosphate-buffered saline (PBS) at the desired density.

### Animals, immunization and infection protocol

Three to four weeks-old female BALB/c and SCID mice were obtained from Charles River (l'Abresle, France) and maintained under specific pathogen-free conditions in the animal facilities. The mice were i.n. immunized as previously described [21], [22]. Briefly, mice (groups of 4–5 mice) were slightly sedated with pentobarbital (CEVA Santé Animale - La Ballastière, France) and inoculated by pipetting 20 µl PBS containing approximately 1×10^6^ CFU of *B. pertussis* BPZE1, *B. pertussis* BPSM or *B. parapertussis* onto the tip of the nares. The mice were sacrificed 7 days after challenge, and their lungs were harvested, homogenized in PBS and plated in serial dilutions onto BG-blood agar to count CFUs after incubation at 37°C for three to four days, as described [21], [22].

### Intracellular staining

For the analysis of intracellular IL-17 and IFN-γ, 2.5×10^6^ cells/ml from naïve or mice immunized 8 weeks previously with BPZE1 were cultured in 48-well tissue culture plates (Nunclon, Roskilde, Denmark) in the presence of 10 µg/ml of heat-inactivated PTX or FHA for 18 to 20 h. PTX was purified from FHA-deficient *B. pertussis* BPGR4 [49] and FHA from PTX-deficient BPRA [50] as described in [51] and [52], respectively. Brefeldin A (One µl of BD GolgiPlug, BD Biosciences) was added to the cultures for the last 5 h to prevent secretion of intracellular cytokines. One million cells were labelled with the FITC-conjugated anti-CD4 antibody (clone H129.19; BD Biosciences) and APC-conjugated anti-CD8 antibody (clone 53-6.7; BD Biosciences) for 30 min at 4°C. Cells were then washed and fixed with BD Cytofix/Cytoperm^TM^ Plus Fixation/Permeabilisation Kit, according to the manufacturer's protocol (BD Biosciences). To label intracellular IL-17 or IFN-γ, cells were incubated with phycoerythrin-conjugated anti-IL-17 (clone TC11-18H10) or anti-IFN-γ antibodies (clone XMG1.2; BD Biosciences) in the presence of saponin for 30 min at 4°C, washed, and acquired on a cytofluorometer (Becton Dickinson). Lymphocytes were gated by their forward and side light-scattering properties, and 100,000 cells were acquired. Analysis was done with the Cell Quest program.

### *In vitro* studies

For *in vitro* proliferation assays, whole spleen cells (1×106) were seeded in U-shaped 96-well culture plates in a final volume of 200 µl RPMI 1640 with 10% FBS (Invitrogen, Carlsbad, California) containing 2 mM glutamine, 100 IU/ml penicillin, 100 µg/ml streptomycin, 10 mM HEPES, 1 mM sodium pyruvate and 50 µM 2-ME, and stimulated for 60 h with FHA (10 µg/ml). Proliferation was assessed by the incorporation of 3[H]TdR during the last 16 h of culture. Results are expressed as mean counts per minute of 3[H]TdR incorporation for triplicate cultures of groups with three to four mice. Culture supernatants were taken after 48 h of stimulation with FHA, and their concentrations of IFN-γ, IL-2, IL-10 and IL-17 were determined by ELISA (R&D systems, Abingdon, U.K.) using manufacturer's specifications. In some experiments, the medium was supplemented with 10 µg/ml blocking anti-IL-10 (clone JES5-2A5; BD Biosciences) or anti-IL-17 (clone TC11-18H10; BD Biosciences) antibodies.

### Cell separation

To enrich for CD4+ and CD8+ T cells, spleen cells were incubated with FITC-conjugated anti-CD4 antibodies (BD Biosciences) or PE-conjugated anti-CD8 antibodies (BD Biosciences), for 30 min at 4°C. After washing, cells were resuspended, and CD4+ or CD8+ T cells were isolated using a cell sorter separation technology (Beckman coulter). The CD4+ and CD8+ cell populations were sorted to a purity of >98% and >99%, respectively.

### Adoptive transfer

To study protection after passive/adoptive transfer, serum (100 µl), whole spleen cells (20 - 50×10^6^) or purified CD4^+^ or CD8^+^ (7.5×10^5^) spleen T cells from non-immunized mice or mice immunized with BPZE1 eight weeks previously were injected intraperitoneally to SCID mice. 24 h later, the mice were infected with *B. pertusssis* BPSM or *B. parapertussis*, and protection was assessed on day 8 as described above.

### Serum-killing assays

Bacteria were grown for 48 h on BG agar and then diluted to 600 CFU/µl in PBS. One microliter was added to 80% mouse serum diluted in PBS, in a final volume of 200 µl. After one h of incubation at 37°C, 100 µl was plated onto BG-blood agar and incubated at 37°C for CFU counting.

### Statistical analysis

Results were analysed using the unpaired Student t test and the ANOVA test (GraphPad Prism program) when appropriate. Differences were considered significant at *p*≤0.05.

## References

[1] Greenberg DP, von König CH, Heininger U (2005). Health burden of pertussis in infants and hildren.. Pediatr Infect Dis J.

[2] McIntyre P, Wood N (2009). Pertussis in early infancy: disease burden and preventive strategies.. Curr Opin Infect Dis.

[3] Von König CH, Halperin S, Riffelmann M, Guiso N (2002). Pertussis of adults and infants.. Lancet Infect Dis.

[4] Crowcroft NS, Stein C, Duclos P, Birmingham M (2003). How best to estimate the global burden of pertussis?. Lancet Infect Dis.

[5] Edwards KM (2005). Overview of pertussis: focus on epidemiology, sources of infection, and long term protection after infant vaccination.. Pediatr Infect Dis J.

[6] Storsaeter J, Wolter J, Locht C (2007). Pertussis vaccines. *In Bordetella* Molecular Microbiology..

[7] Jefferson T, Rudin M, DiPietrantonj C (2003). Systematic review of the effects of pertussis vaccines in children.. Vaccine.

[8] Sato Y, Kimura M, Fukumi H (1984). Development of a pertussis component vaccine in Japan.. Lancet.

[9] Decker MD, Edwards KM (2000). Acellular pertussis vaccines.. Pediatr Clin North Am.

[10] Pichichero ME, Deloria MA, Rennels MB, Anderson EL, Edwards KM (1997). A safety and immunogenicity comparison of 12 acellular pertussis vaccines and one whole-cell pertussis vaccine given as a forth dose in 15- to 20-months-old children.. Pediatrics.

[11] Olin P, Rasmussen F, Gustafsson L, Hallander HO, Heijbel H (1997). Randomised controlled trial of two-component, three-component, and five-component acellular pertussis vaccines compared with whole-cell pertussis vaccine. Ad Hoc Group for the Study of Pertussis Vaccines.. Lancet.

[12] Hellwig SM, van Spriel AB, Schellekens JF, Mooi FR, van de Winkel JG (2001). Immunoglobulin A-mediated protection against *Bordetella pertussis* infection.. Infect Immun.

[13] Letowska I, Hryniewicz W (2004). Epidemiology and characterization of *Bordetella parapertussis* strains isolated between 1995 and 2002 in and around Warsaw, Poland.. Eur J Clin Microbiol Infect Dis.

[14] Parkhill J, Sebaihia M, Preston A, Murphy LD, Thomson N (2003). Comparative analysis of the genome sequences of *Bordetella pertussis*, *Bordetella parapertussis* and *Bordetella bronchiseptica*.. Nat Genet.

[15] Mastrantonio P, Giuliano M, Stefanelli P, Sofia T, De Marzi L (1997). *Bordetella parapertussis* infections.. Dev Biol Stand.

[16] He Q, Viljanen MK, Arvilommi H, Aittanen B, Mertsola J (1998). Whooping cough caused by *Bordetella pertussis* and *Bordetella parapertussis* in an immunized population.. JAMA.

[17] Bergfors E, Trollfors B, Taranger J, Lagergard T, Sundh V (1999). Parapertussis and pertussis: differences and similarities in incidence, clinical course, and antibody responses.. Int J Infect Dis.

[18] Heininger U, Stehr K, Schmitt-Grohe S, Lorenz C, Rost R (1994). Clinical characteristics of illness caused by *Bordetella parapertussis* compared with illness caused by *Bordetella pertussis*.. Pediatr Infect Dis J.

[19] David S, van Furth R, Mooi FR (2004). Efficacies of whole cell and acellular pertussis vaccines against *Bordetella parapertussis* in a mouse model.. Vaccine.

[20] Wolfe DN, Goebel EM, Bjornstad ON, Restif O, Harvill ET (2007). The O antigen enables *Bordetella parapertussis* to avoid *Bordetella pertussis*-induced immunity.. Infect Immun.

[21] Mielcarek N, Debrie AS, Raze D, Bertout J, Rouanet C (2006). Live attenuated *B. pertussis* as a single-dose nasal vaccine against whooping cough.. PLoS Pathog.

[22] Feunou PF, Ismaili J, Debrie AS, Huot L, Hot D (2008). Genetic stability of the live attenuated *Bordetella pertussis* vaccine candidate BPZE1.. Vaccine.

[23] Mills KH (2001). Immunity to *Bordetella pertussis*.. Microbes Infect.

[24] Mahon BP, Sheahan BJ, Griffin F, Murphy G, Mills KH (1997). Atypical disease after *Bordetella pertussis* respiratory infection of mice with targeted disruptions of interferon-gamma receptor or immunoglobulin mu chain genes.. J Exp Med.

[25] Centers for Disease Control and Prevention (2004). Fatal case of unsuspected pertussis diagnosed from a blood culture-Minnesota, 2003.. MMWR Morb Mortal Wkly Rep.

[26] Mills KH, Barnard A, Watkins J, Redhead K (1993). Cell-mediated immunity to *Bordetella pertussis*: role of Th1 cells in bacterial clearance in a murine respiratory infection model.. Infect Immun.

[27] Byrne P, McGuirk P, Todryk S, Mills KH (2004). Depletion of NK cells results in disseminating lethal infection with *Bordetella pertussis* associated with a reduction of antigen-specific Th1 and enhancement of Th2, but not Tr1 cells.. Eur J Immunol.

[28] Kirimanjeswara GS, Mann PB, Harvill ET (2003). Role of antibodies in immunity to *Bordetella infections*.. Infect Immun.

[29] Leef M, Elkins KL, Barbic J, Shahin RD (2000). Protective immunity to *Bordetella pertussis* requires both B cells and CD4 (+) T cells for key functions other than specific antibody production.. J Exp Med.

[30] Menozzi FD, Mutombo R, Renauld G, Gantiez C, Hannah JH (1994). Heparin-inhibitable lectin activity of the filamentous hemagglutinin adhesin of *Bordetella pertussis*.. Infect Immun.

[31] Higgins SC, Jarnicki AG, Lavelle ED, Mills KHG (2006). TLR4 mediates vaccine-induced protective cellular immunity to *Bordetella pertussis*: role of IL-17-producing T cells.. J Immunol.

[32] Chen X, Howard OMZ, Oppenheim JJ (2007). Pertussis toxin by inducing IL-6 promotes the generation of IL-17-producing CD4 cells.. J Immunol.

[33] McGuirk P, McCann C, Mills KH (2002). Pathogen-specific T regulatory 1 cells induced in the respiratory tract by a bacterial molecule that stimulates interleukin 10 production by dendritic cells: a novel strategy for evasion of protective T helper type 1 responses by *Bordetella pertussis*.. J Exp Med.

[34] Mills KHG, Ryan M, Ryan E, Mahon BP (1998). A murine model in which protection correlates with pertussis vaccine efficacy in children reveals complementary roles for humoral and cell-mediated immunity in protection against *Bordetella pertussis*.. Infect Immun.

[35] Watanabe M, Nagai M (2004). Whooping cough due to *Bordetella parapertussis*: an unresolved problem.. Expert Rev Anti-infect Ther.

[36] Watanabe M, M Nagai (2001). Reciprocal protective immunity against *Bordetella pertussis* and *Bordetella parapertussis* in a murine model of respiratory infection.. Infect Immun.

[37] Goebel EM, Wolfe DN, Elder K, Stibitz S, Harvill ET (2008). O antigen protects *Bordetella parapertussis* from complement.. Infect Immun.

[38] Mascart F, Verscheure V, Malfroot A, Hainaut M, Piérard D (2003). *Bordetella pertussis* infection in 2-months-old infants promotes type 1 T cell responses.. J Immunol.

[39] Woolard MD, Hensley LL, Kahula TH, Frelinger JA (2008). Respiratory *Francisella tularensis* live vaccine strain infection induces Th17 cells and prostaglandin E2, which inhibits generation of gamma interferon-positive T cells.. Infect Immun.

[40] Ma CS, Chew GYJ, Simpson N, Priyadarshi A, Wong M (2008). Deficiency of Th17 cells in hyper IgE syndrome due to mutations in *STAT3*.. J Exp Med.

[41] Page G, Sattler A, Kersten S, Thiel A, Radbruch A (2004). Plasma cell-like morphology of Th1-cytokine-producing cells associated with the of CD3 expression.. Am J Path.

[42] Veldhoen M, Hocking RJ, Atkins CJ, Locksley RM, Stockinger B (2006). TGFβ in the context of an inflammatory cytokine milieu supports de novo differentiation of IL-17-prodicing T cells.. Immunity.

[43] Annunziato F, Cosmi L, Stantarlasci V, Maggi L, Liotta L (2007). Phenotypic and functional features of human Th17 cells.. J Exp Med.

[44] Rowan AG, Fletcher JM, Ryan EJ, Moran B, Hegarty JE (2008). Hepatitis C virus-specific Th17 cells are suppressed by virus-induced TGF-beta.. J Immunol.

[45] Nasso M, Fedele G, Spensieri F, Palazzo R, Costantino P (2009). Genetically detoxified pertussis toxin induces Th1/Th17 immune response through MAPKs and IL-10 dependent mechanisms.. J Immunol.

[46] Fedele G, Nasso M, Stensieri F, Palazzo R, Frasca L (2008). Lipopolysaccharides from *Bordetella pertussis* and *Bordetella parapertussis* differently modulate human dendritic cell functions resulting in divergent prevalence of Th17-polarized responses.. J Immunol.

[47] Harrington LE, Hatton RD, Mangan PR, Turner H, Murphy TL (2005). Interleukin 17-producing CD4+ effector T cells develop via a lineage distinct from the T helper type 1 and 2 lineages.. Nat Immunol.

[48] Dubin PJ, Kolls JK (2008). Th17 cytokines and mucosal immunity.. Immunol Rev.

[49] Locht C, Geoffroy MC, Renauld G (1992). Common accessory genes for the *Bordetella pertussis* filamentous hemagglutinin and fimbrae share sequence similarities with the *papC* and *papD* gene families.. EMBO J.

[50] Antoine R, Locht C (1990). Roles of the disulfide bond and the carboxy-terminal region of the S1 subunit in the assembly and biosynthesis of pertussis toxin.. Infect Immun.

[51] Sekura RD, Fish F, Manckark CR, Meade B, Zhang YL (1983). Pertussis toxin. Affinity purification of a new ADP-ribosyltransferase.. J Biol Chem.

[52] Menozzi FD, Gantiez C, Locht C (1991). Interaction of *Bordetella pertussis* filamentous hemagglutinin with heparin.. FEMS Microbiol Lett.

